# A Novel Variant of CORO1A Gene Contributing to the Development of Primary Immunodeficiency in Children

**DOI:** 10.1155/crii/3233892

**Published:** 2026-06-17

**Authors:** Alanoud Aljohani, Yazeed Alayed, Bashayer Alrasheed, Hamza Alghamdi, Mofareh Alzahrani, Khalid Alshaigi

**Affiliations:** ^1^ Pediatric Allergy and Immunology Consultant, Department of Allergy and Immunology, Main Children Hospital, King Fahad Medical City, P.O. Box. 59046, Riyadh, 11525, Saudi Arabia, kfmc.med.sa; ^2^ Pediatric Allergy and Immunology Fellow, Department of Allergy and Immunology, Main Children Hospital, King Fahad Medical City, P.O. Box. 59046, Riyadh, 11525, Saudi Arabia, kfmc.med.sa

**Keywords:** B-cell and CORO1A, severe combined immunodeficiency, T-cell

## Abstract

**Introduction:**

The case report describes a novel finding of a homozygous variant in the coronin 1A (CORO1A) gene, associated with atypical severe combined immunodeficiency (SCID) in a 9‐year‐old female patient with recurrent infections and unique immunological features, including periodic T‐cell lymphocytosis and T‐ and B‐cell lymphopenia.

**Case History and Examination:**

A 9‐year‐old female with a known history of recurrent pneumonia presented to the emergency department with a 2‐week history of intermittent fever, progressive lethargy, and pallor. Her past medical history was remarkable for multiple hospital admissions secondary to community‐acquired pneumonia and urinary tract infections, totaling four admissions to date. Family history was significant for consanguinity between parents and a healthy 4‐year‐old younger male sibling. Chest computed tomography (CT) demonstrated bilateral diffuse centrilobular nodules, scattered ground‐glass opacities, and left lower lobe consolidation, in addition to a tree‐in‐bud pattern. Immunological evaluation revealed T‐cell lymphocytosis, B‐cell lymphopenia, and a decreased CD4/CD8 ratio. Based on these findings, the pediatric allergy and immunology team recommended genetic testing for primary immunodeficiency. The panel identified a homozygous variant of uncertain significance (VUS) in the CORO1A gene. Pathogenic variants in CORO1A are associated with autosomal recessive CORO1A‐related SCID.

**Conclusion:**

The novel homozygous variant in the CORO1A gene suggests the likelihood of an atypical form of SCID, characterized by periodic T‐cell lymphocytosis, T‐cell lymphopenia, B‐cell lymphopenia, and a low CD4/CD8 ratio, expanding the spectrum of CORO1A deficiency.

**Key Clinical Message**

Most severe combined immunodeficiency (SCID) disorders arise from profound defects in T‐cell proliferation and maturation within the thymus, resulting in T‐lymphopenia. Mutations in CORO1A have been strongly associated with the failure of mature T cells to exit the thymus into peripheral circulation, leading to marked T‐cell lymphopenia. Alternatively, peripheral immune defects—such as unresponsiveness to T‐cell receptor (TCR) stimulation or impaired migration to sites of infection—have been hypothesized. In the present case, the presence of T‐cell lymphocytosis makes the latter mechanism more plausible, suggesting that defective T‐cell migration or signaling rather than impaired thymic egress may underlie the observed immune dysfunction.

## 1. Introduction

Severe combined immunodeficiency (SCID) represents a rare group of genetic disorders affecting distinct pathways of the cellular and humoral immune systems. Dysfunction in these pathways renders affected individuals highly susceptible to recurrent and often life‐threatening infections. To date, nearly 20 genes have been identified as potential culprits in SCID pathogenesis [1, 2]. Among these genes, coronin 1A (CORO1A) protein—a member of the coronin family—has been recognized as a cause of autosomal recessive SCID with variable phenotype in the form of T‐cell lymphopenia of variable severity, preserved B, and natural killer cell count [3] 5/7/2026 8:21:00 a.m.

CORO1A protein plays a critical role in actin cytoskeleton regulation by antagonizing actin polymerization and promoting actin severing [4, 5]. The protein’s crown‐like distribution along the cell surface inspired the name “coronin.” It was first identified in the mold *Dictyostelium discoideum* as a soluble protein bound to the actin–myosin complex [6]. CORO1A protein is exclusively expressed in lymphocytes and is inherited in an autosomal recessive pattern [3, 7, 8]. Loss of gene function may result in impaired chemotaxis and phagocytosis [9]. Complete absence of CORO1A expression is associated with SCID phenotype, while hypomorphic mutation might lead to a milder phenotype and late‐onset manifestation [10, 11].

A total of 12 patients have been reported in the literature to date. The first case, described in 2008, presented with (T−, B+, and NK+) SCID secondary to a severe postvaccination varicella infection, along with attention deficit hyperactivity disorder [4, 12]. Subsequently, three consanguineous patients with hypomorphic CORO1A deficiency were reported to develop Epstein–Barr virus (EBV)‐induced B‐cell lymphoproliferation, thereby expanding the clinical spectrum of the disease [10]. Later, a compound heterozygous mutation was identified in association with chronic human papillomavirus infection manifesting as epidermodysplasia verruciformis, accompanied by severe oral herpetic lesions [13]. Another study described a novel mutation causing a mucocutaneous immunodeficiency syndrome characterized by susceptibility to human papillomavirus, herpes simplex virus type 1, and leprosy in siblings with late‐onset disease manifestations [14]. In 2019, the first case of early detection through newborn screening was reported, involving a novel mutation in CORO1A, presenting with only mild lymphopenia and neutropenia and no significant impairment of immune function [15]. More recently, a distinct mutation associated with mild CD4 lymphopenia and recurrent viral infections was described, permitting survival into adulthood [11]. Additionally, a separate mutation was identified in a child presenting with recurrent urinary tract infections, otitis media, and developmental delay, who was successfully treated with hematopoietic stem cell transplantation [16]. Finally, a novel mutation affecting natural killer cell calcium signaling was reported in a child with recurrent infections, disseminated papillomatous disease, ulcerative dermatitis, and autism spectrum disorder, further broadening the phenotypic spectrum associated with CORO1A deficiency [3].

In this case report, we describe a novel homozygous variant in the CORO1A gene associated with an atypical presentation of SCID.

## 2. Case History and Examination

**Figure 1:**
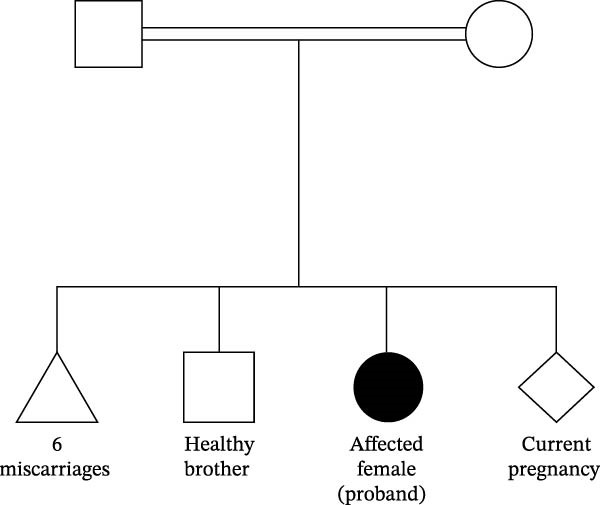
Family pedigree.

A 9‐year‐old female, known to have recurrent pneumonia, presented to the emergency department with a 2‐week history of intermittent fever, lethargy, and pallor. She had initially been admitted to a rural hospital at fever onset, where she was found to have deranged liver function tests. Two days after discharge, her fever recurred, accompanied by abdominal pain, prompting presentation to the King Fahad Medical City emergency department. She also reported night sweats, poor weight gain, and joint pain. Past medical history was significant for recurrent hospital admissions secondary to community‐acquired pneumonia and urinary tract infections, totaling four admissions. Past surgical history was noncontributory. The family history revealed consanguinity between the parents and a healthy 4‐year‐old younger male sibling. The family history showed a consanguineous parent, with a 4‐year‐old younger male sibling who is healthy. On examination, the patient appeared ill‐looking and irritable, with notable drenching sweats. She was vitally stable and afebrile at presentation. No digital clubbing or skin lesions were observed. Cardiopulmonary examination was unremarkable. Abdominal examination revealed mild generalized tenderness without organomegaly. Peripheral lymph nodes were not palpable. Laboratory investigations demonstrated markedly elevated white blood cell counts. She was admitted for inpatient care due to a suspicion of hematological malignancy and to rule out underlying immunodeficiency disorders. Family pedigree is illustrated in Figure 1.

## 3. Investigations, Differential Diagnoses, and Treatment

The laboratory workup is summarized in Table 1. No blast cells were identified on the peripheral blood smear. Abdominal ultrasound revealed mild hepatomegaly without focal lesions. Chest computed tomography (CT) demonstrated bilateral diffuse centrilobular nodules with scattered ground‐glass opacities, left lower lobe consolidation, and a tree‐in‐bud appearance. Blood, urine, and stool cultures were negative. Screening for tuberculosis, including purified protein derivative (PPD), acid‐fast bacilli cultures, and QuantiFERON‐TB testing, yielded negative results. Serological testing for hepatitis B and C viruses, EBV, parvovirus B19, cytomegalovirus, and human immunodeficiency virus was also negative. A lymphocyte subset panel demonstrated T‐cell lymphocytosis, B‐cell lymphopenia, and a reduced CD4/CD8 ratio (Tables 2 and 3). Adenosine deaminase (ADA) activity was within the normal range. Based on these findings, the pediatric allergy and immunology team recommended genetic testing for primary immunodeficiency, which revealed a homozygous variant of uncertain significance (VUS) in the CORO1A gene. Pathogenic variants in this gene are known to cause autosomal recessive CORO1A‐related SCID; however, the clinical relevance of this particular variant remains uncertain. Given the immunological findings, subcutaneous immunoglobulin therapy was initiated at a dosing schedule of every 2 weeks in the daycare unit. Additional imaging included a brain CT scan, which demonstrated bilateral white matter hypodensities, and a paranasal sinus CT scan, which showed nonspecific mucosal inflammatory changes with active secretion in the left maxillary sinus.

**Table 1:** Hematological workup upon initial presentation to ER.

Laboratory workup	Upon initial presentation	Prior to discharge	Upon second admission
White blood cells (4.30–11.30 10^3^/μL)	29.9	8.06	8.61
Absolute neutrophils (1.35–7.50 × 10^3^/μL)	24.3	3.24	2.9
Absolute eosinophils (0.25–1.00 × 10^3^/μL)	0.17	0.15	0.12
Absolute basophils (0.01–0.1 × 10^3^/μL)	0.11	0.08	0.10
Absolute lymphocyte (1.90–4.90 × 10^3^/μL)	2.32	3.67	4.18
Absolute monocytes (0.25–1.00 10^3^/μL)	2.31	0.92	1.31
Hemoglobin (11–15 g/dL)	13.7	12.6	12.5
Platelets (150–450 × 10^3^/μL)	649	525	499
CRP (<5.0 mg/L)	24.3	14.9	

*Note*: Total WBC and differential values are depicted in 10^3^/μL, HGB value is depicted in g/dL, and CRP is depicted in mg/L.

**Table 2:** Immunoglobulin levels with flow cytometric analysis of lymphocyte subsets.

Immunoglobulin subset	Values
IgM (0.43–1.7 g/L)	0.775
IgG (3.5–12.4 g/L)	3.46
IgA (0.40–1.20 g/L)	0.446
IgE	2.31
*Clostridium tetani* toxin IgG	0.070
*Corynebacterium diphtheriae* toxin IgG	0.14

*Note:* Immunoglobulin subset values are expressed in g/L. Vaccination titters are expressed in IU/Ml.

**Table 3:** Lymphocyte markers.

Lymphocytes markers	Initial values at presentation	Interim values after resolution of infection
Absolute lymphocytes count (×10^9^/L)	2.75	0.92
Absolute CD3 (1.70–1.90 × 10^9^/L)	2.37	0.77
CD3 (%)	86.3	83.5
Absolute CD4 (0.80–1.70 × 10^9^/L)	1.05	0.29
CD4 (%)	38.4	31.3
Absolute CD8 (0.7–1.00 × 10^9^/L)	1.2	0.45
CD8 (%)	43.7	49
CD4/CD8 ratio (0.9–1.4)	0.87	0.64
Absolute CD19 (0.40–0.80 × 10^9^/L)	0.23	0.08
CD19 (%)	8.4	8.3
HLA‐DR% on B lymphocytes%	100	100
Absolute CD16+56 (%)	0.099	11.1
CD16+56% (0.20–0.40 × 10^9^/L)	3.6	0.10

*Note:* Total lymphocytes and subsets values are expressed in ×10^9^/L and percentage.

Genetic testing was performed using a targeted next‐generation sequencing (NGS) approach. Genomic DNA was enzymatically fragmented, and regions of interest were enriched using sequence‐specific capture probes designed to target genes associated with immunodeficiency (genes tested are provided in Appendix A). Sequencing was conducted on an Illumina platform with concurrent copy number variation (CNV) analysis of the targeted genes. The assay targets the coding regions of these genes, including ±10 base pairs of flanking intronic sequences as well as known pathogenic or likely pathogenic variants within both coding and selected noncoding regions included in the enrichment design. Data analysis was carried out using validated bioinformatics pipelines. Identified variants were assessed for pathogenicity and clinical relevance and classified according to American College of Medical Genetics and Genomics (ACMG) guidelines. Analytical sensitivity is ≥99.9% for reported variants. Variants of low quality and/or with uncertain zygosity were confirmed using orthogonal methods, such as Sanger sequencing.

## 4. Outcome and Follow‐Up

The identified novel homozygous variant in the CORO1A gene suggests the likelihood of an atypical form of SCID. The patient’s immunological profile—characterized by abnormal T‐cell lymphocytosis, B‐cell lymphopenia, and a reduced CD4/CD8 ratio—points toward a possible defect in T‐cell receptor‐mediated signaling or impaired lymphocyte migration. Target gene parental segregation analysis revealed the same variant coordinate in a heterozygous state for both parents (Table 4). The identified variant in the COROA1 gene is present in the gnomAD database with an allele frequency of nearly 1.3 × 10^−5^, indicating extreme scarcity. Continued follow‐up in the pediatric immunology clinic was arranged to monitor immune function, response to subcutaneous immunoglobulin therapy, and the potential emergence of new clinical manifestations.

**Table 4:** Patient’s genetic result and parental segregation analysis.

	Genome coordinates	Variant coordinates	Amino acid change	Zygosity	Type
Patient	NC_000016.10:g.30187382_30187384del (HG38)	NM_007074.4:c.637_639delGAG	Glu213del	Homozygous	In‐frame deletion ^∗^
Father	NC_000016.10:g.30187382_30187384del (HG38)	NM_007074.4:c.637_639delGAG	Glu213del	Heterozygous	In‐frame deletion ^∗^
Mother	NC_000016.10:g.30187382_30187384del (HG38)	NM_007074.4:c.637_639delGAG	Glu213del	Heterozygous	In‐frame deletion ^∗^

*Note:*  ^∗^The variant might be annotated as a splice junction because it begins at the first nucleotide of a coding exon (c.637). While a nearby variant (c.637−14C>T) is intronic, the position of this variant at the exon boundary raises the possibility of an effect on the splice acceptor site or disruption of exonic splicing enhancers.

## 5. Discussion

The majority of SCID disorders arise from a profound defect in T‐cell proliferation, thymic maturation, or cell survival, leading to T‐cell lymphopenia [17]. According to the most recent diagnostic criteria established by the Primary Immune Deficiency Treatment Consortium (PIDTC) in 2022, typical SCID is defined by very low T‐cell counts (<0.05 × 10^9^/L), whereas atypical SCID is characterized by T‐cell counts ranging from 0.05–1 × 10^9^/L for age, in the presence of a pathogenic genetic variant and after exclusion of other SCID subtypes, combined immunodeficiencies with known genotypes, thymic disorders, or other causes of T‐cell lymphopenia [18]. In our case, the identified variant in the CORO1A together with a postinfectious T‐cell count of ~0.77 × 10^9^/L, is consistent with a diagnosis of atypical SCID according to PIDTC criteria. To our knowledge, this is the first novel report of CORO1A deficiency manifesting as periodic T‐cell lymphocytosis, T‐cell lymphopenia, and B‐cell lymphopenia with low CD4/CD8 ratio, implicating the CORO1A gene.

CORO1A encodes an actin‐regulating protein featuring a β‐propeller domain formed by seven WD40 repeats, which is crucial for cytoskeletal dynamics, together with a C‐terminal coiled domain that mediates oligomerization and protein stability [7, 19].

Traditionally, CORO1A mutations have been strongly associated with impaired egress of mature T cells from the thymus into the peripheral circulation, resulting in profound T‐cell lymphopenia [4, 10]. In addition, defects in T‐cell activation and migration have been described, which may contribute to the variability in phenotype [12, 20]. Establishing a clear genotype–phenotype correlation remains challenging due to the limited number of reported cases, heterogeneity in disease onset, and the expanding mutational spectrum. Severe phenotypes are generally attributed to the complete loss of CORO1A function, whereas milder or atypical presentations are thought to result from hypomorphic variants that allow residual protein activity [21].

Our patient’s novel variant, c.637_639del (p.Glu213del), is the first reported 3‐bp in‐frame deletion within the propeller domain and thereby reinforces that the WD repeats constitute a mutational hotspot. Interestingly, the p.Glu213 residue, that lies within the N‐terminal β‐propeller domain of CORO1A. This domain is highly sensitive to structural perturbations, and even subtle alterations may disrupt protein stability or interaction surfaces without completely abolishing protein expression [7, 19]. Hence, in‐frame deletion of a single amino acid was associated with a hypomorphic variant resulting in a milder phenotype in the form of partial T‐cell defects, expanding the spectrum of COROA1‐related immunodeficiency phenotypic variability.

The majority of reported CORO1A variants cause loss‐of‐function through frameshifts (e.g., c.248_249delCT [p.Pro83ArgfsX10] and c.1077delC [p.Q360RfsX44]) or large deletions [4, 11, 14]. These variants result in the complete absence of functional protein and a severe phenotype dominated by EBV‐driven lymphoproliferation. In contrast, a hypomorphic missense variant (e.g., c.717G >A [p.Val134Met]) allows residual protein expression and function, producing a more variable clinical spectrum that aligns closely with the phenotype observed in our patient [10].

Notably, the c.1077delC variant—described by both Punwani et al. and Mace and Orange et al.—shows complete loss of detectable CORO1A protein yet presents with late disease onset [13, 14]. Intriguingly, Dinur Schejter et al. reported that the c.601C >T (p.Arg201Cys) variant also leads to complete absence of protein by western blot [15], yet the patient exhibited only periodic neutropenia and lymphopenia, suggesting a relatively mild phenotype. This finding challenges the earlier assumption that complete loss‐of‐function is invariably associated with severe early‐onset disease and highlights the ongoing challenges in establishing precise genotype–phenotype correlations for CORO1A deficiency. In keeping with this complexity, Ovadia et al. [16] described the c.602G >A (p.Arg201His) variant affecting the same codon region as reported by Dinur Schejter et al. [15]. Although western blotting demonstrated complete loss of protein expression, it was associated with a severe clinical phenotype.

**Nomenclature**
CT: Computed tomography
HG: Human genome
KFMC: King Fahad Medical City
PIDTC: Primary Immune Deficiency Treatment Consortium
SCID: Severe combined immunodeficiency
TCR: T‐cell receptor.

## Author Contributions

Alanoud Aljohani conceived the case report, collected, and analyzed data. Yazeed Alayed and Mofareh Alzahrani wrote the initial draft of the article and provided logistic support. Khalid Alshaigi and Hamza Alghamdi reviewed the final draft and provided supervision.

## Funding

No funding was received for this research.

## Disclosure

All authors have critically reviewed and approved the final draft and are responsible for the manuscript’s content and similarity index. Authors testify that all persons designated as authors qualify for authorship and have checked the article for plagiarism. If plagiarism is detected, all authors will be held equally responsible and will bear the resulting sanctions imposed by the journal thereafter.

## Ethics Statement

Approval from the Institutional Review Board (IRB) of King Fahad Medical City (KFMC) was obtained (IRB Registration Number with KACST, KSA).

## Consent

Written informed consent was obtained from the legally authorized next of kin to publish the details pertaining to the case report of the subject involved.

## Conflicts of Interest

The authors declare no conflicts of interest.

## Data Availability

The data that support the findings of this study are available upon request from the corresponding author. The data are not publicly available due to privacy or ethical restrictions.

## Appendix A

Genes tested by NGS:

ACD, ACP5, ACTB, ADA, ADA2, ADAM17, ADAR, AICDA, AIRE, AK2, AP1S3, AP3B1, AP3D1, APOL1, ARPC1B, ATM, ATP6AP1, B2M, BACH2, BCL10, BCL11B, BLM, BLNK, BRCA1, BRCA2, BRIP1, BTK, C1QA, C1QB, C1QC, C1R, C1S, C2, C3, C5, C6, C7, C8A, C8B, C8G, C9, CARD11, CARD14, CARD9, CARMIL2, CASP10, CASP8, CCBE1, CCDC39, CCDC40, CCNO, CD19, CD247, CD27, CD3D, CD3E, CD3G, CD40, CD40LG, CD46, CD55, CD59, CD70, CD79A, CD79B, CD81, CD8A, CDC42, CDCA7, CEBPE, CFAP298, CFAP300, CFB, CFD, CFH, CFHR1, CFI, CFP, CFTR, CHD7, CIB1, CIITA, CLCN7, CLPB, COG6, COPA, CORO1A, CR2, CSF2RB, CSF3R, CTC1, CTLA4, CTPS1, CTSC, CXCR2, CXCR4, CYBA, CYBB, CYBC1, DBR1, DCLRE1B, DCLRE1C, DEF6, DIAPH1, DKC1, DNAAF1, DNAAF11, DNAAF19, DNAAF2, DNAAF3, DNAAF4, DNAAF5, DNAAF6, DNAH11, DNAH5, DNAH9, DNAI1, DNAI2, DNAJB13, DNAJC21, DNAL1, DNASE1L3, DNASE2, DNMT3B, DOCK2, DOCK8, DRC1, DRC2, DRC4, DSG1, EFL1, ELANE, EPG5, ERBIN, ERCC4, ERCC6L2, EXTL3, F12, FAAP24, FADD, FANCA, FANCB, FANCC, FANCD2, FANCE, FANCF, FANCI, FANCL, FANCM, FAS, FASLG, FAT4, FCGR3A, FCHO1, FCN3, FERMT1, FERMT3, FNIP1, FOXN1, FOXP3, FPR1, G6PC3, G6PD, GAS2L2, GATA1, GATA2, GFI1, GINS1, GUCY2C, HAX1, HELLS, HMOX1, HYDIN, HYOU1, ICOS, IFIH1, IFNAR1, IFNAR2, IFNGR1, IFNGR2, IGHM, IGLL1, IKBKB, IKZF1, IL10, IL10RA, IL10RB, IL12B, IL12RB1, IL12RB2, IL17F, IL17RA, IL17RC, IL18BP, IL1RN, IL21, IL21R, IL23R, IL2RA, IL2RB, IL2RG, IL36RN, IL6R, IL6ST, IL7R, INO80, IRAK1, IRAK4, IRF2BP2, IRF3, IRF4, IRF7, IRF8, IRF9, ISG15, ITCH, ITGB2, ITK, JAGN1, JAK1, JAK3, KDM6A, KMT2A, KMT2D, KRAS, LAMTOR2, LAT, LCK, LIG1, LIG4, LPIN2, LRBA, LYST, MAD2L2, MAGT1, MALT1, MAP3K14, MASP2, MCIDAS, MCM4, MECOM, MEFV, MOGS, MPO, MS4A1, MSH6, MSN, MTHFD1, MVK, MYD88, MYSM1, NBAS, NBN, NCF1, NCF2, NCF4, NCKAP1L, NCSTN, NFAT5, NFE2L2, NFKB1, NFKB2, NFKBIA, NHEJ1, NHP2, NLRC4, NLRP1, NLRP12, NLRP3, NME8, NOD2, NOP10, NRAS, NSMCE3, OAS1, ODAD1, ODAD2, ODAD3, ORAI1, OSTM1, OTULIN, PALB2, PARN, PAX1, PEPD, PGM3, PIK3CD, PIK3R1, PLCG2, PMM2, PMS2, PNP, POLA1, POLD1, POLD2, POLE, POLE2, POLR3A, POLR3C, POLR3F, POMP, PRF1, PRKCD, PRKDC, PSENEN, PSMB8, PSMG2, PSTPIP1, PTEN, PTPRC, RAB27A, RAC2, RAD51, RAD51C, RAG1, RAG2, RANBP2, RASGRP1, RBCK1, REL, RELA, RELB, RFWD3, RFX5, RFXANK, RFXAP, RHOG, RHOH, RIPK1, RMRP, RNASEH2A, RNASEH2B, RNASEH2C, RNF168, RNF31, RNU4ATAC, RORC, RPSA, RSPH1, RSPH4A, RSPH9, RTEL1, SAMD9, SAMD9L, SAMHD1, SBDS, SEC61A1, SEMA3E, SERPING1, SH2D1A, SH3BP2, SH3KBP1, SKIC2, SKIC3, SLC29A3, SLC35C1, SLC39A4, SLC39A7, SLC46A1, SLC7A7, SLX4, SMARCAL1, SMARCD2, SNX10, SP110, SPAG1, SPINK5, SPPL2A, SRP54, SRP72, STAT1, STAT2, STAT3, STAT5B, STIM1, STING1, STK36, STK4, STN1, STX11, STXBP2, TAFAZZIN, TAP1, TAP2, TAPBP, TBK1, TBX1, TCF3, TCIRG1, TCN2, TERC, TERT, TET2, TFRC, TGFB1, TGFBR1, TGFBR2, THBD, TICAM1, TINF2, TIRAP, TLR3, TLR7, TMC6, TMC8, TNFAIP3, TNFRSF11A, TNFRSF13B, TNFRSF13C, TNFRSF1A, TNFRSF4, TNFRSF9, TNFSF11, TOP2B, TP53, TPP1, TPP2, TRAF3, TREX1, TRNT1, TTC7A, TYK2, UBE2T, UNC13D, UNC93B1, UNG, USB1, USP18, VPS45, WAS, WDR1, WIPF1, WRAP53, XIAP, XK, XRCC2, ZAP70, ZBTB24, ZMYND10, ZNF341, ZNFX1.
